# Life Style and Cancer – The Open Symposium of the Japanese Environmental Mutagen Society (JEMS) in 2015

**DOI:** 10.1186/s41021-016-0036-x

**Published:** 2016-05-01

**Authors:** Hiroyuki Kamiya, Shinji Oikawa

**Affiliations:** Graduate School of Biomedical and Health Sciences, Hiroshima University, 1-2-3 Kasumi, Minami-ku, Hiroshima, 734-8553 Japan; Graduate School of Medicine, Mie University, 2-174 Edobashi, Tsu, Mie 514-8507 Japan

**Keywords:** Cancer, Lifestyle, Intestinal environment, Oral environment, Obesity, Smoking, Infection, Inflammation

## Abstract

The Open Symposium of the Japanese Environmental Mutagen Society (JEMS), entitled “Life Style and Cancer”, was held at the Shiba-Kyoritsu campus of Keio University, Tokyo, on May 30, 2015. The major aim of this symposium was to provide information about the relationships between lifestyle habits and cancers and an opportunity to inform a wide range of people about the JEMS activities.

Neoplasms, cardiac disorders, and cerebrovascular diseases have been the leading causes of death for Japanese people during the last five decades. This fact indicates that disorders originating from bad lifestyles are major contributors to premature death. In particular, death by neoplasms has continually increased. Therefore, the reduction in the number of cancer cases and deaths is an important issue in Japan, and also in other countries.

The Open Symposium of the Japanese Environmental Mutagen Society (JEMS), entitled “Life Style and Cancer”, was held at the Shiba-Kyoritsu campus of Keio University, Tokyo, on May 30, 2015. The major aim of this symposium was to provide information about the relationships between lifestyle habits and cancers and an opportunity to inform a wide range of people about the JEMS activities. During the symposium, six expert scientists made presentations. To provide an overview of cancer causes in Japanese people, we invited Dr. Minako Nagao, a leading scientist in research on the mutagenicity of heterocyclic amines, as a keynote speaker. For the general presentations, focused on recent findings on human cancer causes from a broad perspective, five expert scientists, including non-JEMS members, were invited. These discussions have been summarized within this special collection of *Genes and Environment* by the symposium organizers (Hiroyuki Kamiya and Shinji Oikawa). The symposium’s program is reproduced below:

- Yasunobu Aoki (President, JEMS), Opening Speech
- Hiroyuki Kamiya (Hiroshima University), Introduction

Keynote Lecture (Chair: Yasunobu Aoki)

- Minako Nagao (formerly National Cancer Center, Tokyo), Lifestyle and Cancer: Cancer Causes in Japanese

Session 1 (Chairs: Hiroyuki Kamiya and Masami Yamada)

- Satoshi Matsumoto (Yakult Central Institute), Intestinal Environment and Cancer: Effects of foods and intestinal bacterial flora
- Michihiro Mutoh (National Cancer Center, Tokyo), Obesity and Cancer: Clarification of clinical state towards chemoprevention

Session 2 (Chairs: Shinji Oikawa and Akira Takeiri)

- Yasusei Kudo (University of Tokushima), Oral Environment and Cancer: Mechanism of carcinogenesis influenced by oral environment
- Masahiko Watanabe (Shujitsu University), Smoking and Cancer: Cancer risk estimation and effects of smoking cessation
- Shosuke Kawanishi (Suzuka University of Medical Science), Infection and Cancer: formation of reactive oxygen and nitrogen species by chronic inflammation and its roles in carcinogenesis
- Panel Discussion (Chairs: Hiroyuki Kamiya and Shinji Oikawa)
- Shinji Oikawa (Mie University), Closing Speech

Dr. Minako Nagao summarized the relationships between cancer deaths and population aging, and talked about the concept of “Tenju-gann” or “natural-end cancer” (cancer in people of advanced age that leads to a peaceful death with minimal suffering) proposed by Drs. Kitagawa and Sugimura. The present status in Japan was suggested to be close to the Tenju-gann, but insufficient. She next listed many causes of cancers, including smoking, infection, alcohol consumption, obesity, excessive salt intake, insufficient intake of vegetables and fruits, and lack of exercise. She discussed the possible involvement of heterocyclic amines in foods, the mutagenic compounds that she has studied for many years, in the onset of cancers in the colon and other tissues, based on the literature. She finally referred to the importance of the age factor when the causes of cancers are studied.

Dr. Satoshi Matsumoto first explained the differences in the intestinal bacterial flora in babies and adults, and indicated that certain foods are the major cause of these alterations. Human adult intestinal flora is classified as three enterotypes, by the occupancies of *Bacteroidetes*, *Prevotella*, and *Rumunococcus* bacteria. These enterotypes are considered to be dependent on environmental (foods and social situations) and host (bowel motility and gut immune system) factors. Unhealthy diets and various types of stress may affect the intestinal flora, and thus contribute to carcinogenesis. Therefore, the proper microbial diversity of the intestinal bacterial flora should be maintained, to protect against cancer and other diseases. Dr. Matsumoto showed that certain bacteria in intestines are linked to cancer, and that probiotic products may reduce cancer risk via the control of the intestinal flora.

Dr. Michihiro Mutoh talked about cancers induced by obesity, with a central focus on colon cancer. Obesity increases colon cancer risk, and the important factors are insulin resistance, abnormal lipid metabolism, and imbalance of adipocytokines. The adipocytokines, cytokines secreted by adipose tissue, cause insulin resistance and abnormal lipid metabolism. In addition, they play important roles in inflammation induction. Dr. Mutoh explained that these states correspond to a chronic inflammatory disorder. Thus, proper diet, physical activity, and chemoprevention are pivotal for the prevention of obesity-related cancers. As an example of cancer chemoprevention, he described clinical trials of aspirin for colon cancer prevention.

Prof. Yasusei Kudo demonstrated that the oral environment is related to infectious diseases, diabetes, cardiac diseases, dementia, and cancers. Numerous bacteria are present in the oral cavity, and the normal oral flora acts as a defense mechanism against infection, a source of vitamins, and a developer of lymph tissue. In contrast, periodontopathic bacteria are involved in colon cancer progression and increase the risk of various cancers. Moreover, in addition to smoking and alcohol drinking, oral mucosal diseases due to infection by Candida, human papilloma virus and Epstein-Barr virus cause oral cavity cancers. Therefore, Prof. Kudo emphasized the importance of proper oral care.

Prof. Masahiko Watanabe explained the relationship between tobacco smoking and cancers. Tobacco smoking causes cancers of the lung, mouth, and larynx, which are directly exposed to the smoke, and also induces cancers in many tissues involved in the absorption, distribution, and excretion of components of the smoke. The risks of cancer death for male and female Japanese smokers are 2- and 1.6-fold higher than those for Japanese non-smokers, respectively. He emphasized that smoking cessation reduces cancer risk for the rest of the smoker’s life, and also reduces the risk of death by other diseases.

Prof. Shosuke Kawanishi reported that ~18 % of cancers in the world are caused by infection, and that more than 25 % of cancers are estimated to be induced by inflammation, due to infection plus other diseases such as ulcerative colitis. The formation of DNA damage is pivotal in the carcinogenic pathways triggered by infection and inflammation. He described the formation of the mutagenic 8-nitroguanine base from guanine, by the reactive nitrogen and oxygen species in inflamed tissues, and its roles in carcinogenesis. Prof. Kawanishi finally concluded that 8-nitroguanine is a novel and useful biomarker for carcinogenesis related to inflammation.

There were ~130 participants at the symposium. The questionnaire survey revealed that more than 40 % of the participants were not members of the Japanese Environmental Mutagen Society. The responses to the questionnaire included many positive comments; for example, “The symposium was excellent since the theme was immediate” and “the symposium was very useful.” Based on these responses to the questionnaire, we feel that the symposium was a favorable experience for the participants. We would like to take this opportunity to express our thanks to everyone involved with this symposium.

